# Collagenase-3 (MMP-13) deficiency protects C57BL/6 mice from antibody-induced arthritis

**DOI:** 10.1186/ar4423

**Published:** 2013-12-27

**Authors:** Anjana Singh, Narendiran Rajasekaran, Bettina Hartenstein, Sibylle Szabowski, Mieczyslaw Gajda, Peter Angel, Rolf Bräuer, Harald Illges

**Affiliations:** 1 Department of Natural Sciences, Immunology and Cell Biology, University of Applied Sciences, von-Liebig-Straße 20, Rheinbach D-53359, Germany; 2 Present address: Department of Pathology, Maastricht University (CARIM), P. Debyelaan 25, Maastricht 6229 HX, the Netherlands; 3 Present address: Pediatric Immunology, CCSR Room No. 2120, 269 Campus Drive, Stanford, CA 94305, USA; 4 Division of Signal Transduction and Growth Control, DKFZ-ZMBH Alliance, Deutsches Krebsforschungszentrum, Heidelberg D-69120, Germany; 5 University Hospital Jena, Institute of Pathology, Ziegelmühlenweg 1, Jena D-07740, Germany

## Abstract

**Introduction:**

Matrix metalloproteinases (MMPs) are important in tissue remodelling. Here we investigate the role of collagenase-3 (MMP-13) in antibody-induced arthritis.

**Methods:**

For this study we employed the K/BxN serum-induced arthritis model. Arthritis was induced in C57BL/6 wild type (WT) and MMP-13-deficient (MMP-13^**–/–**^) mice by intraperitoneal injection of 200 μl of K/BxN serum. Arthritis was assessed by measuring the ankle swelling. During the course of the experiments, mice were sacrificed every second day for histological examination of the ankle joints. Ankle sections were evaluated histologically for infiltration of inflammatory cells, pannus tissue formation and bone/cartilage destruction. Semi-quantitative PCR was used to determine MMP-13 expression levels in ankle joints of untreated and K/BxN serum-injected mice.

**Results:**

This study shows that MMP-13 is a regulator of inflammation. We observed increased expression of MMP-13 in ankle joints of WT mice during K/BxN serum-induced arthritis and both K/BxN serum-treated WT and MMP-13^**–/–**^ mice developed progressive arthritis with a similar onset. However, MMP-13^**–/–**^ mice showed significantly reduced disease over the whole arthritic period. Ankle joints of WT mice showed severe joint destruction with extensive inflammation and erosion of cartilage and bone. In contrast, MMP-13^**–/–**^ mice displayed significantly decreased severity of arthritis (50% to 60%) as analyzed by clinical and histological scoring methods.

**Conclusions:**

MMP-13 deficiency acts to suppress the local inflammatory responses. Therefore, MMP-13 has a role in the pathogenesis of arthritis, suggesting MMP-13 is a potential therapeutic target.

## Introduction

There is a growing body of evidence implicating matrix metalloproteinases (MMPs) as major players in numerous disease conditions including atherosclerosis, tumor invasion, ulcerative diseases and arthritic diseases [1-4]. Rheumatoid arthritis (RA) is a chronic arthritic disease resulting in joint destruction and loss of function in the joints. Articular cartilage degradation, characteristic of RA, is believed to be mediated by the collagenase subfamily of MMPs [5]. Collagenases cleave fibril collagens at neutral pH and play an important role in matrix remodeling. Collagens are the major structural proteins of all connective tissues. The most abundant collagens are types I, II and III, called interstitial collagens. Type I collagen is widely distributed, being produced in bone, skin, tendons, and ligament, whereas type II collagen is located almost exclusively in hyaline cartilage.

Collagenase-3/MMP-13 is the most recently identified member of the collagenase subfamily, originally isolated from breast carcinoma [6]. In addition to its expression in breast tumors, MMP-13 mRNA exhibits a more restricted pattern of expression within connective tissue, and is usually found in articular cartilage [7], in bone [8] and in chondrocytes in osteoarthritis (OA) [9-11]. Moreover, MMP-13 was found in the synovial tissue from patients with OA or RA [12]. MMP-13 was found to degrade collagen types I, II and III and the cartilage proteoglycan aggrecans [13]. Biochemical characterization of MMP-13 revealed a broad spectrum of activities against connective tissue components [14]. In light of the preference of MMP-13 for collagen type II of hyaline cartilage degrading this substrate more efficiently as compared with MMP-1 and MMP-8 [14], one is tempted to speculate that MMP-13 is a critical component of the cellular machinery executing the turnover of articular cartilage, thus highlighting this molecule as a potential therapeutic target for treatment of cartilage destruction. Indeed, Li and colleagues recently described the inhibition of MMP-13 as a new hope for the treatment of OA [15]. Pharmaceutical inhibition of MMP-13 resulted in reduced arthritis in the collagen-induced arthritis and severe combined immunodeficiency mouse coimplantation model, but not in the antibody-induced arthritis model [16].

In this study we investigated the role on MMP-13 in the K/BxN sera-transfer arthritis model. In the K/BxN model, arthritis occurs spontaneously in those mice expressing both the transgene-encoded KRN T-cell receptor and the IA^g7^ major histocompatibility complex class II allele [17,18]. These transgenic T cells are specific for a self-peptide derived from the glycolytic enzyme glucose-6-phosphate isomerase (GPI) and are able to break tolerance in the B-cell compartment, resulting in the production of autoantibodies to GPI [19-21]. Joint specificity is explained by the deposition of the GPI onto the articular cartilage surface, binding of anti-GPI antibodies to the surface and subsequent complement-mediated inflammation [22]. Transfer of serum from the K/BxN mice into C57BL/6 mice resulted in the development of a transient arthritis in the recipients. Here we show that MMP-13 expression is increased in C57BL/6 mice during the course of the K/BxN serum-induced arthritis and that mice deficient for MMP-13 (MMP-13^–/–^) are protected from inflammation and joint destruction.

## Methods

### Experimental animals

KRN T-cell receptor-transgenic mice on the C57BL/6 background were a kind gift from Dr D Mathis and Dr C Benoist (Strasbourg, France) and were bred to NOD/Lt (N) mice to generate K/BxN mice [17]. MMP-13^–/–^ mice on a pure C57BL/6 background were described previously [23]. C57BL/6 wild-type (WT) mice were obtained from Charles River Laboratories (Sulzfeld, Germany). All mice were maintained and bred at the animal facility of the University of Applied Sciences, Rheinbach. All mice were sex and age-matched for experiments and were between 6 and 10 weeks of age. All experiments were approved by the Nordrhein-Westfalen state authorities (Landesamt für Natur und Umweltschutz) and complied with European Community regulations (86/609/EEC) for the care and use of laboratory animals.

### Arthritic serum preparation and transfer into mice

Serum was separated from the blood obtained by bleeding from the tail vein of arthritic K/BxN mice (12 to 18 weeks old), pooled and frozen at –20°C until use. For inducing arthritis in WT or MMP-13^–/–^ mice, each mouse was injected intraperitoneally with 200 μl equivalent of serum from arthritic K/BxN mice.

### Assessment of antibody-induced arthritis by ankle thickness and clinical index score

Development of arthritis in mice was assessed by caliper measurement (model number 33185; Hann &Kolb, Stuttgart, Germany) of ankle thickness and by the clinical index, a visual score based on the number of ankles affected. Ankle thickness for each mouse was expressed in millimeters. The error was expressed as the standard error of the means. The ankle score was 0 if no ankle was affected, and was 1 if one ankle was slightly affected. An inflammation score of 0 was counted if no ankle was affected; a score of 0.25 if an ankle showed reddening and was less swollen; a score of 0.75 if reddening and a moderately swollen ankle was observed; and a score of 1 if an ankle showed reddening and was strongly swollen. Histology was performed and evaluated in a blinded manner as described elsewhere [24].

### RNA extraction

Hind-paw ankle joints from WT mice were isolated at days 0 (that is, control), 3, 8 and 14 after K/BxN serum transfer. The ankle joints were trimmed of skin, muscle, and excess bone around the articular joint and immediately frozen on dry ice. Total RNA was extracted using the PeaqGold total RNA kit (PeaqLab, Erlangen, Germany), according to the manufacturer’s instructions.

### Standard reaction for MMP-13

cDNA products (2 μl) were applied for amplification with 10 units of Taq polymerase and corresponding buffer (Qiagen GmbH, Hilden, Germany) in the presence of 15 mM MgCl_2_, 25 mM dNTP and 5 pmol sense and antisense primers (MWG Eurofins, Ebersberg, Germany). Murine ribosomal large protein P 0 (m36b4) transcript levels served as a quantity and quality control. The reaction was carried out in the PTC-200 system (MJ Research,Biorad, Munich, Germany) with a first cycle of 2.5 minutes at 95°C for complete denaturation, followed by 25 cycles of denaturation (1 minute at 90°C), annealing (1 minute at 61°C for MMP-13 and 1 minute at 60°C for m36b4, respectively) and synthesis (1 minute at 72°C). The conditions were chosen so that the polymerase chain reaction products were in the exponential phase of amplification. Each set of reactions always included a no-sample negative control (water) and a positive control (knee joint of control mouse).

### Primer selection

The following primers were used: MMP-13, sense 5′-CCTTCTGGTCTTCTGGCACAC-3′ and antisense 5′-GGCTGGGTCACACTTCTCTG-3′; and m36b4, sense 5′-AAC ATG CTC AAC ATC TCC CC-3′ and antisense 5′-CCG ACT CCT CCG ACT CTT C-3′.

### Acquisition of gel images and quantitative analysis

Images of reverse transcriptase polymerase chain reaction ethidium bromide-stained agarose gels were acquired with PeqLab E-box (Metabion, Planegg, Germany) and quantification of bands was performed using a Chemidoc (Biorad, Munich, Germany). Band intensity was expressed as relative absorbance units. The sample RNA to be determined and m36b4 was calculated to normalize for initial variation in the sample concentration and as a control for reaction efficiency.

### Statistical analysis

Statistical significance was analyzed using Graphpad Prism software, La Jolla, USA. Data are expressed as the mean ± standard error of the means. Comparisons were performed using the unpaired Student *t* test or by analysis of variance. For comparison of histological data, the nonparametric Mann–Whitney *U* test was used. Differences were considered statistically significant when *P* <0.05.

## Results

### Quantification of MMP-13 mRNA expression in ankle joints of wild-type mice

**Figure 1:**
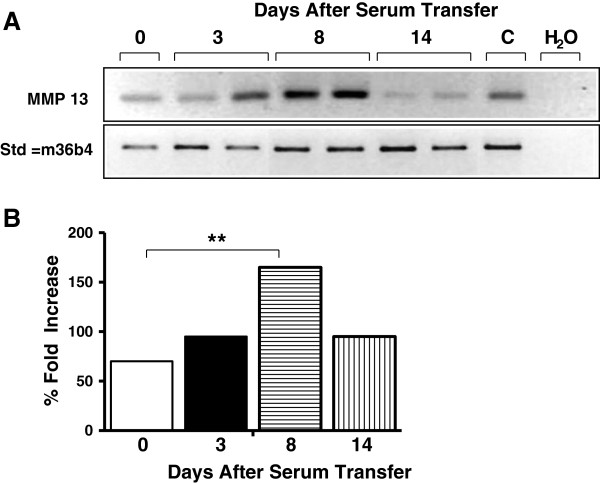
**MMP-13 expression in joints during arthritis development. (A)** Semi-quantitative polymerase chain reaction analysis of RNA derived from hind paws of mice at days 0, 3, and 8 and 14 showing matrix metalloproteinase (MMP)-13 expression (320 base pairs) induced by injection of arthritogenic serum in control C57BL/6 mice. C, cDNA from RNA isolated from mouse bone serving as positive control; m36b4, ribosomal protein PO transcript levels served as control for quality and quantity of tested RNA. **(B)** Quantification of polymerase chain reaction amplified MMP-13 mRNA. **P <0.01.

**Figure 2:**
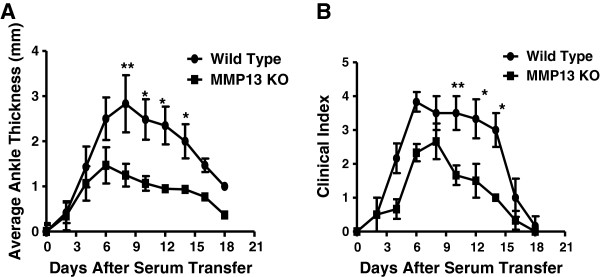
**Mice deficient for MMP-13 were protected from antibody-induced arthritis.** Mice deficient for matrix metalloproteinase-13 (MMP-13^–/–^; *n* = 21) and wild-type mice (*n* = 20) were treated with 200 μl K/BxN sera, and arthritis was evaluated by measuring **(A)** the ankle thickness and **(B)** the clinical index. There was a significant difference in ankle thickness and clinical index scores between the two groups. Values represent the mean ± standard deviation of ankles/time points, which were compared using the Student *t* test. *P <0.05, **P <0.01. KO, knockout.

**Figure 3:**
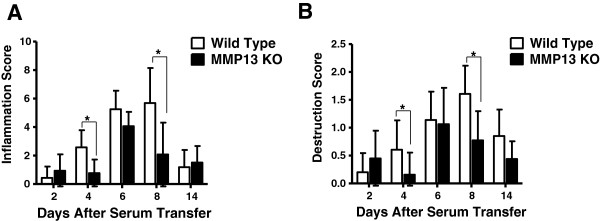
**Arthritis scores of wild-type C57BL/6 and mice deficient for MMP-13 after transfer of K/BxN serum. (A)** Inflammation (infiltration of the synovial tissue by polymorphonuclear mononuclear leukocytes and exudation of granulocytes, hyperplasia of lining cells) and **(B)** cartilage damage in mice was induced with K/BxN sera in wild-type mice and mice deficient for matrix metalloproteinase-13 **(**MMP-13^–/–^). The histology scores for synovial inflammation, cartilage and bone destruction were significantly lower in MMP-13^–/–^ mice compared with the C57BL/6 mice (*P* <0.05). Bars show the mean ± standard deviation. *P* values determined using the Mann–Whitney test (*n* = 3 per genotype per time point). KO, knockout.

To determine whether the expression of MMP-13 was upregulated during arthritis development, we examined its expression in the joints by polymerase chain reaction (Figure 1a). WT mice were injected intraperitoneally with 200 μl arthritogenic K/BxN serum at day 0. Mice were sacrificed and RNA was prepared from the joint tissue, as explained in Methods, at various time points post injection. Semi-quantitative polymerase chain reaction analysis showed that MMP-13 expression increased on day 3, and was highest on day 8 after arthritis induction. Expression on day 14 was reduced to levels seen on day 0. The increase in MMP-13 expression correlated positively with the increase in ankle measurement (Figure 2) and histological scores (Figure 3) observed in mice after arthritis induction.

### MMP-13^–/–^ mice show mild levels of K/BxN serum-induced arthritis

To determine the role of MMP-13 during the development of K/BxN sera-induced arthritis, MMP-13^–/–^ mice and the WT control mice were injected intraperitoneally with 200 μl arthritogenic K/BxN serum and indicative parameters of arthritis were compared in WT mice and mutant mice over time. Arthritis was monitored every 2 days by measuring ankle thickness and clinical index. The onset of arthritis occurred 2 days after injection of sera in both WT control mice and MMP-13^–/–^ mice. The ankle thickness and clinical index increased to a peak value between days 8 and 10, after which the disease resolved until day 18 (Figure 2a,b). However, in contrast to WT mice, MMP-13^–/–^ mice showed reduced arthritis severity with average ankle thickness and clinical index decreased by 1.4-fold (*P* <0.001) and 1.5-fold (*P* <0.001) respectively when compared with the WT mice. These findings suggest that MMP-13 has a significant role in antibody-induced arthritis.

### Decreased inflammation and joint destruction in MMP-13^–/–^ mice

**Figure 4:**
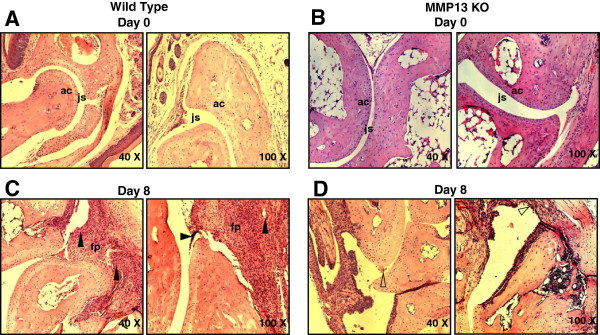
**Representative histopathology of ankle joints from wild-type mice and mice deficient for MMP-13. (A)**, **(B)** Day 0 (before serum transfer): notice the even and clear joint space (js) and smooth articular cartilage (ac). **(C)**, **(D)** Day 8 (post serum transfer): **(C)** wild-type joints display the fibrovascular synovial and periarticular proliferation (fp), erosion of ac, intra-articular exudates and osteolysis in severely affected joints (arrows), whereas **(D)** joints of mice deficient for matrix metalloproteinase-13 (MMP-13) are nearly free of inflammation and the synovium retains its relatively acellular composition (arrows). Images were taken at 40× and 100× magnification. KO, knockout.

Inflammation and joint destruction were assessed histologically from ankle sections of mice sacrificed on days 0, 2, 6, 8 and 14. On day 8 post serum transfer, WT mice showed proliferating inflamed synovial tissue (pannus) expanded over the surface of the articular cartilage (Figure 4c). At sites of contact of the pannus tissue with the cartilage matrix and/or bone, degradation of the cartilage and erosion of the bone surface occurred, whereas in MMP-13^–/–^ mice there was no cartilage destruction or pannus formation observed (Figure 4d). Histological evaluation of synovial inflammation revealed increased infiltration by polymorphonuclear and mononuclear leukocytes, exudation of granulocytes, hyperplasia of the synovial tissue (Figure 3a) and cartilage and bone destruction of joints in WT mice compared with MMP-13^–/–^ mice at days 4 and 8 post serum transfer (Figure 3b). These observations indicate that MMP-13 promotes antibody-induced joint inflammation and destruction.

Clinical severity of arthritis was higher in WT mice compared with MMP-13^–/–^ mice (day 8 (mean ± standard deviation): inflammation score, 6 ± 0.5 in WT mice and 1.8 ± 0.5 in MMP-13^–/–^ mice, *P* <0.05; destruction score, 1.5 ± 0.5 in WT mice and 0.6 ± 0.5 in MMP-13^–/–^ mice, *P* <0.0043) (Figure 3).

## Discussion

The degradation of articular cartilage is a characteristic of RA. Since MMPs are extremely efficient in cleaving cartilage components, they have been implicated in the pathogenesis of RA. The collagenase MMP subfamily members MMP-1 and MMP-13 have both been suggested to play a role in this degradation. Multiple studies have demonstrated a prominent role for MMP-13 in tissue injury and repair. In addition, MMP-13 was recently shown to have a higher activity than MMP-1 against type II collagen [14], which is the major component of articular cartilage. MMP-13 mRNA has been detected in human articular cartilage [7] and synovial membranes [12]. Moore and colleagues found induction of MMP-13 in RA synovial fibroblasts [25].

To understand better the role of MMP-13 in arthritis development and progression we studied the role of MMP-13 in the K/BxN serum-induced arthritis model. We induced arthritis using the K/BxN serum in the MMP-13^–/–^ mice and in the control C57BL/6 mice. Both the control C57BL/6 mice and the MMP-13^–/–^ mice developed arthritis within 2 days after K/BxN serum transfer (Figure 2). However, as the disease progressed the MMP-13^–/–^ mice exhibited a significantly reduced disease when compared with that observed in the C57BL/6 mice. Further, the MMP13^–/–^ mice showed a significant reduction in inflammation and in bone and cartilage destruction when compared with the control C57BL/6 mice on days 4 and 8 post serum transfer (Figure 3). On analyzing the expression of MMP-13 mRNA levels in the joints of the C57BL/6 mice injected with K/BxN serum, we observed a significant increase in the expression of MMP-13 on day 8 post serum transfer when the mice exhibited maximum increase in ankle thickening (Figure 1). These results strongly implicate MMP-13^–/–^ as an important protease in the pathology of antibody-induced arthritis in mice. However, the mild pannus formation and cartilage destruction observed in the MMP-13^–/–^ mice might be due to compensation by other proteases such as cathepsin K, which have been shown to be involved in joint destruction in the collagen-induced arthritis model [26]. The data presented in this study thus suggest, for the first time, an important role for MMP-13 in the progression of antibody-induced arthritis.

Our results correspond well to studies in other mouse models that have implicated MMP-13 in development of arthritis. Recently, Little and colleagues showed that global knockout of MMP-13 could prevent articular cartilage erosion [27]. Jungel and colleagues found upon oral application of a highly selective MMP-13 inhibitor that arthritis was significantly decreased in the severe combined immunodeficiency and collagen-induced arthritis model, whereas no significant effects were seen in the antibody-induced arthritis model [16]. Interestingly, when Wang and colleagues induced OA by meniscal ligamentous injury in MMP-13^Col2ER^ mice, where MMP-13 was specifically knocked out in the cartilage, they observed a significant deceleration in OA progression that was accompanied by a reduced proteoglycan and chondrocyte destruction when compared with OA observed in the control mice [28]. Their results show that a deficiency of MMP-13 in chondrocytes is sufficient for protection against OA. Using the K/BxN serum-transfer we show an increase in expression of MMP-13 on day 8 post serum transfer in the C57BL/6 mice. We also observed a significant reduction in cartilage and bone destruction on day 8 post serum transfer in these mice, indicating a similar role for chondrocyte-specific MMP-13 in antibody-induced arthritis.

In this study we tested the role of MMP-13 using MMP13^–/–^ mice in an antibody-induced arthritis model. Our studies clearly show that deletion of MMP-13 attenuates arthritis progression in mice. MMP-13 inhibition may thus have potential for the treatment of arthritis progression, and the K/BxN serum-induced arthritis model will serve as a useful tool to study the efficiency of MMP-13 inhibitors in treating arthritis.

## Conclusion

This study clearly shows that MMP-13 promotes inflammation and joint destruction in the K/BxN serum-induced arthritis model. Our data demonstrate that MMP-13 is required for a sustained inflammatory response that occurs in the effector phase of arthritis and suggest a role for MMP-13 apart from cartilage destruction in modulating the inflammatory response. Drugs aiming to inhibit MMP-13 function might thus be potential candidates for RA therapy.

## Abbreviations

GPI: Glucose-6-phosphate isomerase; MMP: Matrix metalloprotease; MMP-13–/–: Deficient for MMP-13; OA: Osteoarthritis; RA: Rheumatoid arthritis; WT: Wild type.

## Competing interests

The authors declare that they have no competing interests.

## Authors’ contributions

AS and NR performed animal experiments and data analysis. HI, PA and RB contributed to the design of experiments and interpretation of results. BH, SS and PA performed the joint semi-quantitative study. AS, RB and MG prepared joint specimens and performed the joint histological examination. SS, NR, PA, RB and HI contributed to writing the manuscript. All authors read and approved the final manuscript.
